# Hierarchical Spatial-Spectral Feature Extraction with Long Short Term Memory (LSTM) for Mineral Identification Using Hyperspectral Imagery

**DOI:** 10.3390/s20236854

**Published:** 2020-11-30

**Authors:** Huijie Zhao, Kewang Deng, Na Li, Ziwei Wang, Wei Wei

**Affiliations:** 1 School of Instrumentation Science and Opto-Electronic Engineering, Beihang University, Beijing 100191, China; hjzhao@buaa.edu.cn (H.Z.); dengkewang@buaa.edu.cn (K.D.); ZB1917007@buaa.edu.cn (Z.W.); 2 Beijing Mechanical and Electrical Engineering Design Institute, Beijing 100191, China; cnkicpb@sina.com

**Keywords:** feature extraction, mineral identification, hyperspectral imagery, convolutional neural network (CNN), long short term memory (LSTM)

## Abstract

Deep learning models are widely employed in hyperspectral image processing to integrate both spatial features and spectral features, but the correlations between them are rarely taken into consideration. However, in hyperspectral mineral identification, not only the spectral and spatial features of minerals need to be considered, but also the correlations between them are crucial to further promote identification accuracy. In this paper, we propose hierarchical spatial-spectral feature extraction with long short term memory (HSS-LSTM) to explore correlations between spatial features and spectral features and obtain hierarchical intrinsic features for mineral identification. In the proposed model, the fusion spatial-spectral feature is primarily extracted by stacking local spatial features obtained by a convolution neural network (CNN)-based model and spectral information together. To better exploit spatial features and spectral features, an LSTM-based model is proposed to capture correlations and obtain hierarchical features for accurate mineral identification. Specifically, the proposed model shares a uniform objective function, so that all the parameters in the network can be optimized in the meantime. Experimental results on the hyperspectral data collected by the Airborne Visible/Infrared Imaging Spectrometer (AVIRIS) in the Nevada mining area show that HSS-LSTM achieves an overall accuracy of 94.70% and outperforms other commonly used identification methods.

## 1. Introduction

Minerals tend to form unique spectra due to their distinct constituents [1], and the corresponding regional characteristics are formed during the process of mineralization [2]. With the development of hyperspectral remote sensing, the spectral resolution and spatial resolution have become higher and higher, so lots of novel mineral identification methods have been proposed [3,4]. Traditional mineral identification methods are mainly aimed at the spectra of minerals, including methods based on spectral similarity and methods based on spectral characteristics [5,6,7]. The mineral identification methods based on spectral similarity establish standards to measure the similarity between the identified spectrum and a reference spectrum. Spectral libraries are established to store spectra of various minerals under different conditions for reference, such as the Jet Propulsion Laboratory (JPL) spectral library and United States Geological Survey (USGS) spectral library [8,9]. The spectral angle mapper (SAM) takes the angle between the target spectral vector and the reference spectral vector in the multidimensional space as the criteria to evaluate the similarity [10,11], compared with the spectral correlation mapper (SCM) with the Pearsonian correlation coefficient [12,13] and the spectral information divergence (SID) with the relative information entropy [14,15]. Resemblances between some minerals and differences in the same mineral induce misidentification, and selections of reference spectra of different minerals under various conditions bring challenges to the identification methods based on spectral similarity. Minerals generally present diagnostic absorption bands due to the differences in their respective ions and groups [16]. It is possible to identify minerals by extracting spectral characteristics of the diagnostic absorption bands. In order to better extract the characteristics, continuum removal (CR) is usually performed on the spectrum to enhance and normalize absorption features [17,18]. There exist several common absorption characteristics including absorption depth, absorption width, absorption area, absorption position and absorption symmetry [19]. In addition, the spectral absorption index (SAI) is an absorption characteristic based on spectral diagnostic absorptions to determine the mineral composition and corresponding contents [20]. However, a single absorption characteristic cannot describe adequate features of a diagnostic absorption band, so multiple absorption characteristics are combined to explain the absorption features of minerals accurately and further improve mineral identification accuracy [21]. The relative absorption band-depth (RBD) method produces an RBD image formed of the sum of several channels near absorption shoulders divided by the sum of several channels located near the corresponding absorption valley [22]. Due to various conditions of hyperspectral imaging, spectral absorption features of minerals are easily affected. On the other hand, features of a single absorption band cannot describe the complete features of minerals. 

In recent years, deep learning has made great achievements in imagery analysis, especially in feature extraction [23,24,25]. It usually captures abstract features through a multi-layer network, especially the convolutional neural network (CNN), which is able to extract local spatial features of images through convolutional filters [26,27]. A small number of training samples in the specific dataset are selected to fine tune the parameters, which produces deep learning models more resistant to interference from external factors [28]. More and more researchers in remote sensing are applying deep learning to classification of hyperspectral imagery, making full use of both spatial and spectral features and improving the classification accuracy [29,30,31]. Mei et al. presented a novel five-layer CNN that integrated the spectrum of a pixel and the contextual features of the pixel’s surrounding area as input such as the mean and standard deviation of each band to fulfil classification of hyperspectral imagery [32]. However, too few spatial statistic features were utilized to obtain enough complete features. In [33], a spectral-spatial feature based classification (SSFC) that stacked the spectral features extracted with a balanced local discriminant embedding algorithm and the spatial features extracted with a CNN model was proposed for hyperspectral image classification. However, spatial features and spectral features were extracted and trained separately so that there was not a uniform objective function to optimize them. Zhang et al. proposed a recurrent neural network (RNN)-based model to obtain high-level spatial features from combing low-level features, including texture and differential morphological profiles [34]. However, spectral features were not taken into consideration to obtain spatial-spectral features. Hu et al. proposed a convolutional long short term memory (ConvLSTM) method to extract more discriminative spatial-spectral features, which replaced the full connection layer between the input layer and the hidden layer in long short term memory (LSTM) with the convolution layer [35]. However, the method mainly extracted the correlations in the spatial domain, and less attention was paid to the spectrum of the identified pixel, which could not obtain correlations between the spatial features and the spectral features. Xu et al. proposed a spectral-spatial unified network (SSUN) that extracted spectral features with a LSTM-based model and spatial features with a multiscale CNN (MSCNN) [36]. Then, the spectral features and the spatial features were concatenated to extract the spectral-spatial feature through fully connections. However, correlations between spatial features and spectral features were not further explored to obtain hierarchical features. Moreover, Li et al. proposed a multi-scale CNN to extract multi-scale spectral-spatial features, follow by a LSTM-based model to capture correlations for hierarchical feature extraction [37]. However, the method strengthened the correlations between features maps in different scales, and the pixels in the neighborhood were equally important as input, which greatly reduced the attention to the identified pixel.

To overcome the aforementioned drawbacks, the hierarchical spatial-spectral feature extraction with LSTM (HSS-LSTM) method is proposed to extract hierarchical spatial-spectral features in this paper, which considers correlations between primary spatial features and spectral features. The main contributions of this paper can be summarized as follows: (1) To obtain local spatial features, a CNN-based model is constructed to extract local spatial features of a pixel from the first several principle components of the pixel’s neighborhood region. (2) To construct the primary fusion spatial-spectral feature, the local spatial features of the pixel and its spectrum are stacked together. (3) To reveal the correlations between the components of the fusion feature and obtain intrinsic features, an LSTM-based model is established to refine deep hierarchical spatial-spectral features.

The remainder of this paper is organized as follows. In Section 2, the structure of HSS-LSTM is introduced in detail. Experimental results and corresponding analyses are presented in Section 3. Section 4 provides the conclusions.

## 2. Proposed HSS-LSTM Framework

**Figure 1:**
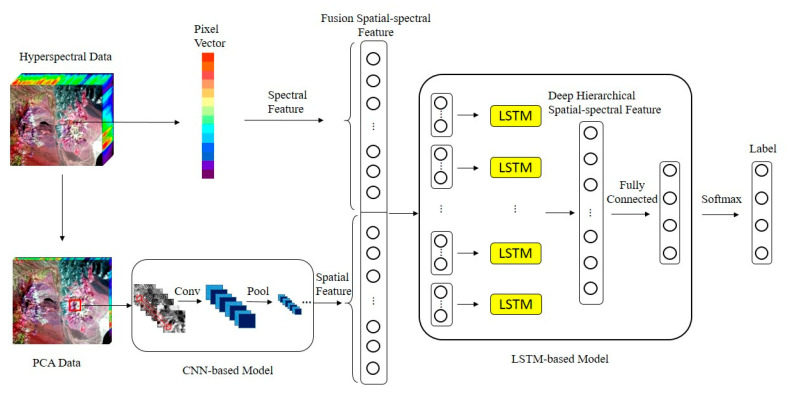
Architecture of HSS-LSTM for mineral identification using hyperspectral data.

The architecture of HSS-LSTM for mineral identification using hyperspectral imagery is shown in Figure 1. Overall, there are three main steps in the proposed model. First, local spatial features are extracted with a CNN-based model which consists of several convolution layers, max pooling layers and a fully connected layer. Then, a fusion spatial-spectral feature is presented which stacks the spectrum of a pixel with its local spatial features. Finally, an LSTM-based model is established to further extract the deep hierarchical spatial-spectral feature from the fusion feature, followed by a softmax layer to fulfil mineral identification.

### 2.1. Spatial Features with CNN-Based Model

**Figure 2:**
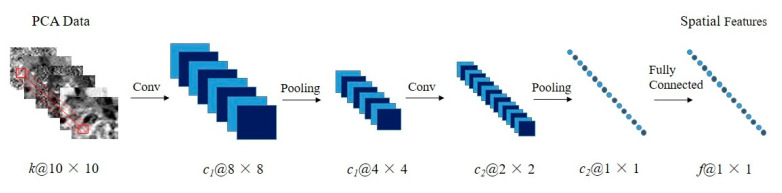
Framework of the proposed CNN-based model for spatial feature extraction. The illustration below each data shows the dimension of the data. For example, a@m × n indicates that the data has channels, and the height and width of the data are m and n, respectively.

CNN is a special neural network which connects the local receptive field with convolution filters, so that it is able to extract local spatial features from images. Generally, a typical CNN model is usually composed of several pairs of convolutional layers and max pooling layers followed by a few fully connected layers [38]. Since the pixels which are closer to the identified pixel have greater influences on its local spatial features, a 10 × 10 neighborhood region of the identified pixel is taken as the input data of the proposed CNN-based model. To obtain spatial features, the proposed CNN-based model consists of two convolution layers with the kernel size of 3 × 3, two max pooling layers with the kernel size of 2 × 2 and a fully connected layer, as shown in Figure 2.

Considering that hyperspectral imagery has hundreds of bands, it leads to redundant information and intensive calculations [39]. Principal components analysis (PCA) is introduced to reduce the dimensionality of the raw hyperspectral data in the spectral domain, while maintaining the spatial information of the data in the meantime [40]. The first *k* principle components, whose eigenvalues account for more than 99% of all the principle components, are fed into the proposed CNN-based model as input [41].

As shown in Figure 2, the proposed CNN-based model has five layers, and the output of the *l*th layer is denoted as *H^l^*, where l∈{1,2,3,4,5}. In addition, *H*^0^ is denoted as the input data. On the condition that the *l*th layer is a convolution layer, a 2-D convolution operation is conducted on *H^l^* to capture features and form a set of feature maps, named as *H^l^*. The process can be formulated as:

(1) $$H^{l}=\sigma (H^{l-1}*W_{Cl}+b_{Cl})$$

where σ(⋅) denotes the activation function, and ∗ denotes the convolution operation. In addition, *W_Cl_* and *b_Cl_* represent the weight matrix and the bias vector that connect *l*th layer and its previous layer, respectively.

When the *l*th layer is a max pooling layer, the data is compressed by replacing the neighborhood region of 2 × 2 with the maximum value of the region. Thus, the nonlinearity of the model is improved. The process is formulated as:

(2) $$H^{l}=\mathrm{pool}(H^{l-1})$$

where pool(·) denotes the max-pooling operation.

At the end of the proposed CNN, a fully connected layer is presented to extract the spatial features denoted as *F_saptial_*. Mathematically, the step can be written as:

(3) $$F_{spatial}=H_{5}=\sigma (W_{F}H_{4}+b_{F})$$

where *W_F_* and *b_F_* represent the weight matrix and the bias vector that connect the fifth layer and the fourth layer.

In order to accelerate the convergence of the proposed model, the sigmoid function is selected as the activation function.

In the proposed CNN-based model, the spatial feature vector of a pixel denoted as $F_{spatial}=\{p_{1},p_{2},\ldots ,p_{f}\}$, which has *f* elements with values between 0 and 1, is extracted from the first several components of its 10 × 10 neighborhood region.

### 2.2. Fusion Spatial-Spectral Feature

Since various minerals present certain characteristics in spectral domain and spatial domain in hyperspectral imagery, it is crucial to capture both spectral features and spatial features when identifying minerals accurately. The spectrum of a pixel contains complete information in spectral domain, which can provide sufficient spectral features for mineral identification. Let $F_{spectral}=\{a_{1},a_{2},\ldots ,a_{b}\}$ denote the spectrum of a pixel with *b* bands, and the fusion spatial-spectral feature *F* of the pixel is constructed by stacking its spatial feature vector *F_saptial_* with the spectral vector *F_spectral_*, which can be written as:

(4) $$F=[F_{spatial},F_{spectral}]=\{p_{1},p_{2},\ldots ,p_{f},a_{1},a_{2},\ldots ,a_{b}\}$$

Due to the fact that the fusion spatial-spectral feature *F* concatenates the spatial features and the spectral features primitively without considering correlations between them, it is necessary to extract deep hierarchical features to achieve high-efficiency mineral identification.

### 2.3. Hierarchical Spatial-Spectral Feature with LSTM-Based Model

Although the fusion feature incorporates the spectral features and the spatial features, there is still a lack of exploration on their correlations to obtain hierarchical features for accurate mineral identification. Unlike other neural networks, RNN is able to process sequential inputs, and the output of the latter subsequence is related to the previous ones due to the connections between their hidden units [42]. In this manner, RNN is generally applied to the situations that data is interrelated, especially sequential data.

**Figure 3:**
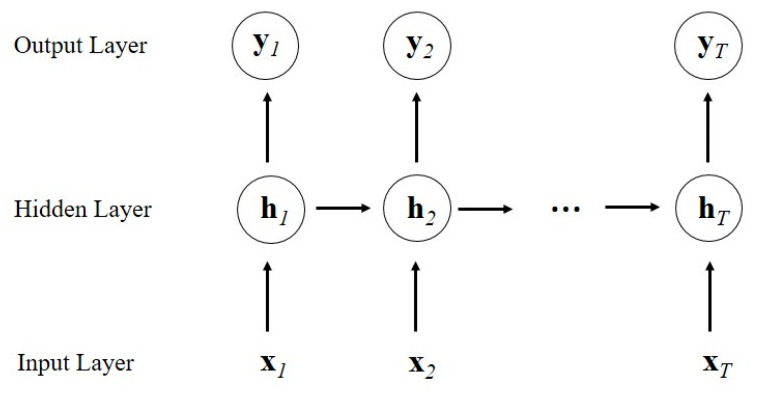
Framework of a simple RNN model.

As shown in Figure 3, for a simple RNN model, the input whose length is *T* can be denoted as $X=\{x_{1},x_{2},\ldots ,x_{T}\}$, and each item x*_t_*, where t∈{1,2,…,T}, is a subsequence. At time step *t*, given the previous hidden layer state *h_t-1_*, the hidden layer state *h_t_* and the output layer state *y_t_* of the current subsequence x*_t_* can be calculated as:

(5) $$h_{t}=\sigma_{R}(W_{h}x_{t}+U_{h}h_{t-1}+b_{h})$$

(6) $$y_{t}=\sigma_{R}(W_{o}h_{t}+b_{o})$$

where *W_h_* and *U_h_* represent the input-to-hidden and the hidden-to-hidden weight matrices respectively, and *W_o_* denotes the hidden-to-output weight matrix. In addition, *b_h_* and *b_o_* denote the input-to-hidden and the hidden-to-output bias vectors separately, and $\sigma_{R}$ denotes the activation function, which is generally the tanh function.

**Figure 4:**
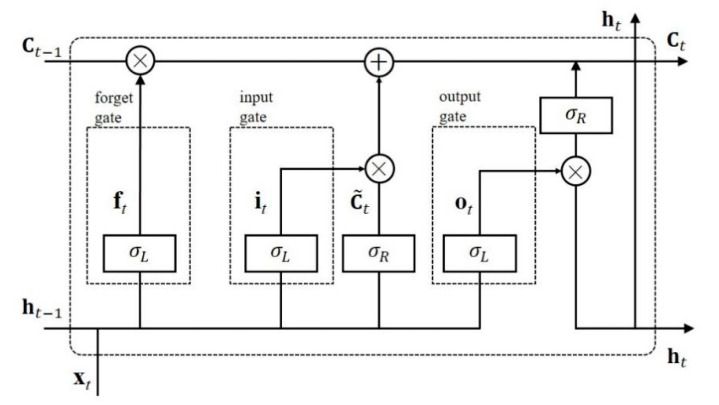
A basic LSTM block. $f_{t}$, $i_{t}$ and $o_{t}$ are outputs of the forget gate, the input gate and the output gate respectively at time step *t*. $C_{t-1}$ and $C_{t}$ correspond to the cell state of the time *t* and time *t-1*. ${\tilde{C}}_{t}$ represents the fresh information brought in at time step *t*.

In order to tackle vanishing gradient and exploding gradient problems in a simple RNN, a variant of RNN is proposed, namely long short-term memory (LSTM) [43,44]. In LSTM, the sigmoid function $\sigma_{L}$ is used as the activation function of three gates, which determine the information transmitted in the network and preserve the errors that can be back-propagated through sequences, as shown in Figure 4.

Compared with a simple RNN, LSTM introduces cell state dominated by three gates with the sigmoid function as the activation function to generate outputs between 0 and 1, which is used to determine how much information retained from the previous steps. The states of the three gates are calculated as:

(7) $$f_{t}=\sigma_{L}(W_{f}\cdot [h_{t-1},x_{t}]+b_{f})$$

(8) $$i_{t}=\sigma_{L}(W_{i}\cdot [h_{t-1},x_{t}]+b_{i})$$

(9) $$o_{t}=\sigma_{L}(W_{o}\cdot [h_{t-1},x_{t}]+b_{o})$$

where *W_f_*, *W_i_* and *W_o_* denote the weight matrices between the input x*_t_* and the forget gate, the input gate and the output gate, respectively. Moreover, *b_f_*, *b_i_* and *b_o_* correspond to the bias vectors of the three gates.

The cell state $C_{t}$ is updated by adding in new information ${\tilde{C}}_{t}$ and discarding part of the information memorized in cell state $C_{t-1}$ as follows:

(10) $${\tilde{C}}_{t}=\sigma_{R}(W_{C}\cdot [h_{t-1},x_{t}]+b_{C})$$

(11) $$C_{t}=i_{t}⊙{\tilde{C}}_{t}+f_{t}⊙C_{t-1}$$

where *W_C_* and *b_C_* correspond to the weight matrix and the bias vector between the input layer and the hidden layer. ⊙ denotes an elementwise multiplication.

The hidden state *h_t_* at time step *t* is determined by o*_t_* and *C_t_* written as:

(12) $$h_{t}=o_{t}⊙\sigma_{R}(C_{t})$$

**Figure 5:**
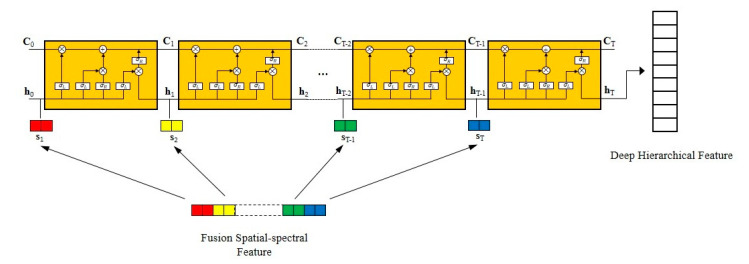
Hierarchical spatial-spectral feature from the fusion feature with the LSTM-based model.

It is obvious that the hidden state *h_t_* incorporates the effective information of the previous *t* time steps in LSTM. In view of the specialty, an LSTM-based model is constructed to extract the deep hierarchical feature from the fusion spatial-spectral feature, as shown in Figure 5. Considering that the proposed LSTM-based model takes sequential data as input, the fusion feature *F* needs to be preprocessed. *F* contains a total of f+b items and is equally divided into *T* dices, so each dice constructs a subsequence of length l=(f+b)/T. In order to facilitate subsequent calculations, *l* needs to be an integer, and *F* is denoted as:

(13) $$F=\{s_{1},s_{2},\ldots ,s_{T}\}$$

where $s_{i},i\in \{1,2,\ldots ,T\}$ is a vector of *l* elements.

Since the fusion spatial-spectral feature *F* is decomposed into *T* subsequences, each subsequence contains partial features and correlates with others. Under the control of the input gate and the forget gate, the cell state $C_{t}$ of the LSTM-based model is able to select the last cell state $C_{t-1}$ and the current subsequence $s_{t}$ to remain the effective features of the first *t* subsequences and abandon the redundant features. The output gate refines $C_{t}$ to acquire the hidden unit *h_t_*, which not only integrates the effective features contained in these *t* subsequences, but also removes the excrescent parts.

As above, with the continuous extension of the proposed LSTM-based model, a growing number of features are stored in the cell state. As for the last subsequence $s_{T}$, its hidden state *h_T_* captures all of the effective features in the fusion spatial-spectral feature *F*. In this way, *h_T_* thinks about correlations between the components of the fusion feature *F* and refines valid features, so it is regarded as the deep hierarchical spatial-spectral feature. In order to fulfil mineral identification, a fully connected layer is added at the end of the proposed LSTM-based model to generate an output with length *n*, followed by a softmax layer to form an intuitive probability distribution of the *n* classes of identified minerals. 

Unlike other models that extract spectral features and spatial features separately resulting in their losses composed of multiple parts, HSS-LSTM refines the stacked spatial-spectral feature to achieve the hierarchical spatial-spectral feature, so that the loss of the model is exclusive. Considering that the LSTM-based model is able to backpropagate the loss primely, the front CNN-based model of HSS-LSTM can also be well trained to extract effective spatial features. To train the parameters in HSS-LSTM, the cross entropy loss is selected as the loss function, and it is minimized by using the adaptive moment estimation (Adam) with back-propagation [45]. After training and fine-tuning HSS-LSTM, the deep hierarchical feature incorporates enough spatial-spectral features which can be used for accurate mineral identification, and the class label for each pixel can be eventually acquired.

## 3. Experimental Results and Analysis

In this section, a well-known hyperspectral dataset which is gathered by the Airborne Visible/Infrared Imaging Spectrometer (AVIRIS) sensor is employed to evaluate HSS-LSTM, namely the Cuprite mine in Nevada, USA. The study area is located on the eastern edge of Esmeralda and Nye County, Nevada (37°29′ to 37°35′ North, 117°9′ to 117°17′ West) [46]. The Nevada mining dataset is covered by various minerals including silicate minerals such as kaolinite and muscovite, sulfate minerals such as alunite, and carbonate minerals such as calcite [47]. The ground truth map of the dataset was produced by the Tricorder 3.3 software [9]. Several experiments are conducted to verify the parameter settings of the HSS-LSTM method and compare with other mineral identification methods. 

### 3.1. Experimental Data

**Figure 6:**
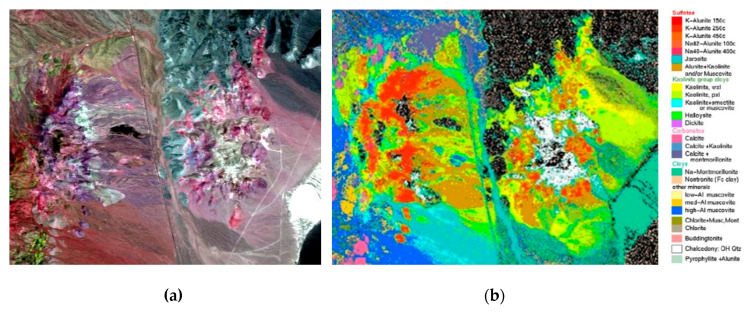
Dataset of the Cuprite mine in Nevada: (**a**) false color image and (**b**) ground truth map.

The Nevada mining dataset consists of 400 × 350 pixels and 50 bands with the wavelength range from 1990 nm to 2480 nm, considering that the spectral features of minerals are mainly concentrated in the short-wave infrared [48]. The false color image and the ground truth map for the dataset are presented in Figure 6.

As shown in Figure 6b, similar minerals that belong to diverse classes thanks to the differences in metal ingredients tend to be merged into the same class. To obtain sufficient training samples and testing samples, and to avoid mixed pixels due to the restriction of the spatial resolution and mineralization, seven classes of widely distributed minerals are selected to be identified, including muscovite, halloysite, calcite, kaolinite, montmorillonite, alunite and chalcedony. Training samples and testing samples are randomly chosen according to the ground truth map, as shown in Table 1.

### 3.2. Analysis of Parameter Settings

HSS-LSTM is mainly constructed with an LSTM-based model, and there are considerable parameters to be configured that are closely related to the efficiency of the method. The PCA data and the original hyperspectral data are preprocessed to restrict their values between 0 and 1, so that HSS-LSTM is able to converge rapidly. As for the CNN-based model, the first three principle components which contain more than 99% information of the hyperspectral imagery are fed in as input. Considering that the sizes of feature maps decrease with the progress of convolution and max pooling, so more feature maps are demanded to maintain information of the data, the sizes of feature maps in different layers are set as $f=c_{2}=2c_{1}$. With a view to the number of spectral bands, *c_1_* is set to 10, 15 and 20 separately, and *f* and *c_2_* are set to 20, 30 and 40 correspondingly. In the LSTM-based model, it takes a sequence of length f+b as input, where *b* is 50, and each subsequence possesses *l* elements. *l* is set to 5 and 10 respectively. The number of the hidden units denoted as *h* is also a significant parameter in the proposed LSTM-based model, which is set to 40, 50 and 60 respectively. To achieve HSS-LSTM for mineral identification, diverse parameter combinations are trained with the learning rate set to 0.0005 and the training epochs set to 1000, and the corresponding overall accuracy (OA) are shown in Table 2.

In Table 2, the overall accuracy is the maximum identification accuracy that can be achieved when HSS-LSTM with the specific parameter combination tends to be stable. It is obvious that the overall accuracy of HSS-LSTM varies with different parameter combinations, and it presents a growth as values of the parameters augment. The reason for this phenomenon is that more features are extracted and maintained when *f*, *l* and *h* are larger. The overall accuracy reaches the peak value when *f*, *l* and *h* are 40, 10 and 50, respectively, and the specific parameter combination is empirically chosen in the following discussion.

### 3.3. Identification Results of the Nevada Dataset

**Figure 7:**
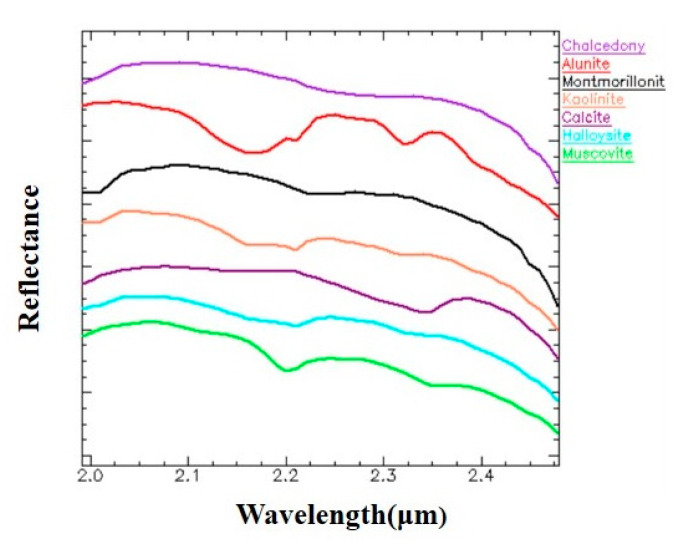
Reference spectra of various minerals.

For evaluating the performance of HSS-LSTM, the identification results of the proposed method are compared with traditional identification methods and other deep learning methods. Specifically, the SAM method is a typical traditional method that utilizes the angle between the spectrum of the identified pixel and the reference spectrum of minerals in the multidimensional space [10]. The average spectrums of training samples of various minerals are taken as reference, as shown in Figure 7. Then the angles between the identified pixel and the reference spectrums are calculated, and the pixel will be classified into the class with the minimum value. In addition, an LSTM-based model with the mere spectrum divided into subsequences with a length of 10 as input is constructed for comparison to explore how spatial features affect the identification accuracy [43]. Besides, a 3D-CNN model is proposed to verify the necessity of deep hierarchical spatial-spectral feature extraction for mineral identification. The 3D-CNN utilizes 3-D filters to convolve the raw hyperspectral dataset [49], and both spectral features and spatial features are extracted in the meantime. The 3D-CNN model has a similar CNN-based network with HSS-LSTM, except that the kernel sizes of the convolution layers and the max pooling layers are 3 × 3 × 11 and 2 × 2 × 2 respectively. The identification accuracies of the test samples over different identification methods are reported in Table 3.

**Figure 8:**
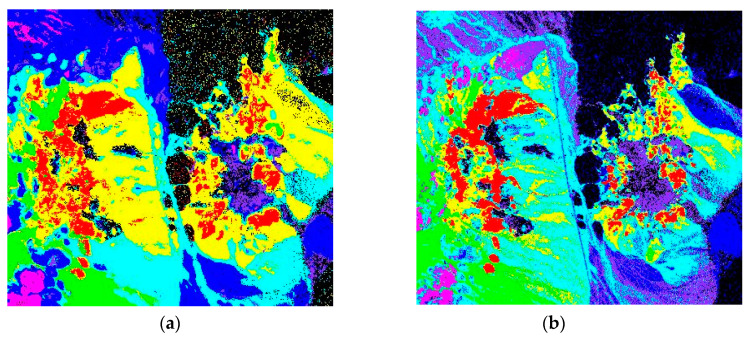

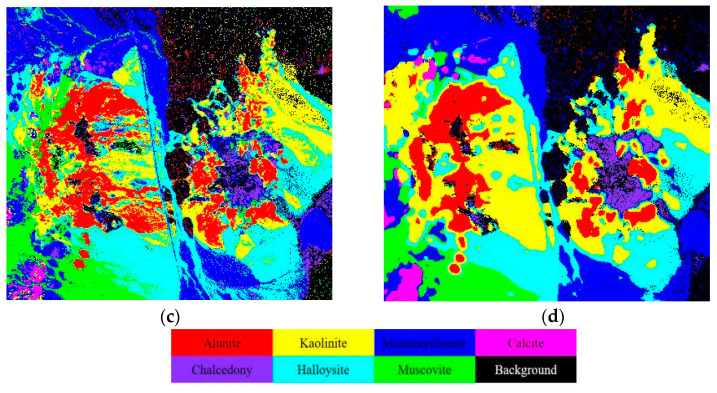
Identification results of the Nevada mining area. (**a**) HSS-LSTM, (**b**) SAM, (**c**) LSTM and (**d**) 3D-CNN.

The UA, PA and AA denote the user’s accuracy, the producer’s accuracy and the average accuracy respectively in Table 3. The HSS-LSTM method achieves rather satisfied identification accuracies of various minerals, and it is obviously superior to other identification methods in terms of the average accuracy and the overall accuracy. To facilitate further discussion, the identification maps of the four different identification methods are shown in Figure 8. The black part of the results indicates the background.

### 3.4. Discussion

According to the experimental results presented above, it is proved that HSS-LSTM achieves better overall and average identification results compared with SAM, LSTM and 3D-CNN. Therefore, the proposed HSS-LSTM method is conducive to promoting mineral identification using hyperspectral imagery. In addition, some further discussions are presented below.

First, due to the similarity between the reference spectrums of halloysite and kaolinite as shown in Figure 7, it is hard to discriminate them in spectral domain. There exist similar problems in [48], where the producer’s accuracy of kaolinite is only 68.4% based on the Kruse and Perry’s (K&P) method. The specific identification results of halloysite and kaolinite on testing samples are presented in Table 4. A large number of testing samples of halloysite are misidentified into kaolinite with SAM and 3D-CNN. The LSTM-based methods including HSS-LSTM and LSTM capture correlations between spectral features and obtain intrinsic features, which promote the overall accuracy of halloysite and kaolinite by more than 10%. More importantly, HSS-LSTM takes correlations between spectral features and spatial features into consideration, which further improves nearly 3.6% overall accuracy compared with LSTM. Besides, it is worth noting that the HSS-LSTM method can significantly improve the producer’s accuracy of kaolinite, reaching 89.50%, which is 15−30% higher than other methods.

Second, the reference spectrum of chalcedony possesses no distinct spectral features, such as significant absorption bands, which results in relatively low identification accuracy with SAM and LSTM. HSS-LSTM improves more than 4% identification accuracy of chalcedony by exploiting spatial features with a CNN-based model. Although 3D-CNN also extracts spectral features and spatial features, HSS-LSTM refines the spatial-spectral feature for the hierarchical feature, which prompts the proposed model to perform the best when identifying chalcedony.

Third, it is noted that the identification map of HSS-LSTM achieves more accurate identification results and reveals more precise textural information according to the ground truth map, as shown in Figure 8. SAM and LSTM only consider the spectrums of each pixel, so it is difficult to form regional structural characteristics. Besides, 3D-CNN lacks further refinement of the spectral-spatial feature, which induces the rough texture of the identification map.

Finally, with the high spectral resolution of hyperspectral remote sensing, a large number of researchers focused on the intrinsic spectral features of minerals to fulfil mineral identification, who conducted experiments on the Nevada mining dataset. The band selection (BS) method achieved an overall accuracy of 87.86% [50], compared with the spectral feature fitting (SFF) method 81.43% [6]. It is obvious that the overall accuracy of 94.70% with the HSS-LSTM method is much better than the identification methods merely based on the spectral features. Although some researchers applied CNN-based models to the mineral identification using hyperspectral images obtained by a hyperspectral camera [23,51], there exist few applications of deep learning in mineral mapping of airborne hyperspectral images. Moreover, the researchers discovered that the spatial features extracted by most of deep learning methods only focus on the geological features without considering correlations with the minerals [52], which highlights the innovation of the proposed HSS-LSTM method.

As above, the HSS-LSTM method achieves better mineral identification results on the experimental dataset by exploring the correlations between spectral features and the spatial features to obtain the hierarchical feature, especially when minerals have similar spectrums or do not have distinct spectral features. In addition, the identification map which is obtain by the proposed method is more consistent with the ground truth. 

## 4. Conclusions

In this paper, in order to better exploit the spectral features and the spatial features of hyperspectral imagery for efficient mineral identification, the HSS-LSTM method is proposed to explore the correlations and obtain the hierarchical spatial-spectral feature. The proposed method exploits a CNN-based model to extract local spatial features followed by an LSTM-based model to capture the correlations between the spatial features and the spectral features in the spectrum, which promotes the identification accuracy of minerals. Besides, the proposed method can backpropagate the loss properly, so all the parameters are able to be optimized in the meantime. Comparative experiments are conducted on the Nevada mining dataset. Experimental results show that the HSS-LSTM method outperforms traditional mineral identification methods and other deep learning methods.

However, the parameters in the HSS-LSTM method affect the identification results greatly, which result in an overall accuracy alteration of more than 3% as shown in Table 2. It is necessary to further optimize them for a better parameter combination. In the other hand, some other state of the art deep learning models are able to extract more efficient spatial features than the proposed CNN-based model. Therefore, our future work will mainly focus on optimizing the parameters of the HSS-LSTM method and extracting spatial features with high-efficiency deep learning models.

## Figures and Tables

**Table 1:** Number of training samples and testing samples in the Nevada dataset.

Class Name	Training Samples	Testing Samples
Muscovite	100	400
Halloysite	100	240
Calcite	100	240
Kaolinite	100	400
Montmorillonite	100	400
Alunite	100	400
Chalcedony	100	240
Total	700	2320

**Table 2:** Identification results with different parameter combinations.

Parameter Combinations ID	*f*	*l*	h	OA (%)
1	20	5	40	91.12
2	20	5	50	91.25
3	20	5	60	91.59
4	20	10	40	91.59
5	20	10	50	91.90
6	20	10	60	92.16
7	30	5	40	91.55
8	30	5	50	92.36
9	30	5	60	92.76
10	30	10	40	92.58
11	30	10	50	92.97
12	30	10	60	93.36
13	40	5	40	93.62
14	40	5	50	94.05
15	40	5	60	94.09
16	40	10	40	93.36
17	40	10	50	94.70
18	40	10	60	94.52

**Table 3:** Identification accuracies of different identification methods on the Nevada dataset.

	HSS-LSTM		SAM		LSTM		3D-CNN	
	UA (%)	PA (%)	UA (%)	PA (%)	UA (%)	PA (%)	UA (%)	PA (%)
Muscovite	98.47	96.50	93.90	100.00	93.90	100.00	100.00	100.00
Halloysite	83.40	87.92	57.78	91.25	68.20	92.92	60.17	88.75
Calcite	100.00	100.00	100.00	92.50	100.00	78.33	100.00	100.00
Kaolinite	82.87	89.50	91.57	57.00	89.67	73.75	90.72	66.00
Montmorillonite	100.00	100.00	96.62	100.00	91.53	100.00	99.26	100.00
Alunite	99.73	91.75	100.00	99.50	99.49	97.25	100.00	100.00
Chalcedony	100.00	97.92	96.98	93.75	100.00	92.50	100.00	96.67
AA (%)	94.80		90.57		90.68		93.06	
OA (%)	94.70		90.17		91.25		92.63	

**Table 4:** Identification results of halloysite and kaolinite on testing samples. A/B denotes that the identification result is A, while the ground truth is B.

Method	Kaolinite/ Kaolinite	Halloysite/ Kaolinite	Kaolinite/ Halloysite	Halloysite/ Halloysite	OA (%)
HSS-LSTM	211	29	41	359	89.06
SAM	219	20	151	228	69.84
LSTM	216	23	56	331	85.47
3D-CNN	213	27	136	264	74.53
